# The influence of commuting time on students’ academic performance and its internal mechanism: an empirical analysis based on CEPS data

**DOI:** 10.3389/fpsyg.2026.1672841

**Published:** 2026-02-06

**Authors:** Ke Shan

**Affiliations:** College of Teacher Education, Quzhou University, Quzhou, China

**Keywords:** academic performance, commuting mode, commuting time, learning engagement, psychological fatigue

## Abstract

**Background:**

This study examines the relationship between commuting time and students’ academic performance, further explores the impact of commuting time on academic performance and its underlying mechanisms, and analyzes strategies to mitigate the negative effects of commuting time on student academic performance.

**Methods:**

The data source for this study is the 2014–2015 China Education Panel Survey (CEPS). This study first employs linear regression to analyze the impact of commuting time on students’ academic performance (Chinese, mathematics, and English scores). It then utilizes the PROCESS procedure to examine the mediating effects of psychological fatigue and learning engagement between commuting time and academic performance. Finally, an interaction term between commuting time and commuting mode is constructed, and hierarchical regression is applied to test the moderating effect of commuting mode on the relationship between commuting time and students’ academic performance.

**Results:**

(1) According to linear regression findings, each additional minute of commuting time correlates with a 0.04-point decrease in Chinese scores, a 0.032-point decline in math scores, and a 0.05-point drop in English scores. (2) The negative impact of commuting time on students’ academic performance exhibits significant variation based on individual student and family factors. (3) Learning engagement and psychological fatigue partially mediate the relationship between commuting time and student performance. (4) Commuting mode significantly moderates the impact of commuting time on students’ academic performance.

**Conclusion:**

Commuting time significantly impedes students’ academic performance. Learning engagement and psychological fatigue partially mediate the relationship between commuting time and students’ academic performance in this population. Commuting mode significantly moderates the effect of commuting time on students’ academic performance. Compared to passive commuting modes, active commuting intensifies the negative impact of commuting time on academic performance. To mitigate the negative effects of commuting time on students’ academic performance, school administrators should optimize campus locations, implement proximity-based enrollment policies, enhance boarding services, and provide psychological counseling and academic support to students.

## 1 Introduction

With the intensification of urbanization in China, the scale of urban space has rapidly expanded, leading to increased time and energy expenditure during commuting. The 2025 commuting monitoring report for major cities in China states that 77% of commutes in major urban areas last less than 45 min, while the proportion of cities with extreme commuting times exceeding 60 min continues to rise. The report also highlights that the prolonged commuting times in large cities have become a key factor affecting individual well-being and work performance.

Due to economic disparities or limited school accommodation services, students choose various commuting methods such as cycling, taking public transportation, using ferries, or walking to school. Commuting has thus become an important part of students’ daily lives. In China, most middle school students prefer to live at home and commute daily between their homes and schools. Commuting saves students accommodation costs and also allows them to benefit from more parental companionship, a wider variety and more nutritious food, better rest spaces, and academic support (Abbasi et al., 2023).

However, many Chinese parents, teachers, and school administrators not only overlook the economic and time costs associated with increased commuting time but also fail to recognize the potential negative impacts of commuting time on students’ mental health (anxiety, depression, and stress), academic performance, attendance, and overall well-being (Chairassamee et al., 2024). In the PISA assessments, Chinese students have consistently outperformed their peers from other countries in reading, mathematics, and science; however, their levels of happiness and life satisfaction are relatively modest, indicating a need for effective interventions from both schools and families. Existing evidence suggests that reducing commuting time positively influences adult job satisfaction, professional identity, and mental health (He et al., 2021; Miao and Wang, 2021). Nonetheless, whether reducing commuting time can enhance the well-being and academic performance of Chinese students remains inconclusive.

Empirical studies indicate that prolonged commuting significantly affects academic performance. Research by Guan et al. (2025) reveals that for every additional hour of commuting time, students’ academic performance declines significantly, and they face higher psychological health risks. Students’ academic performance are influenced not only by family economic status and social class but also closely correlate with the educational environment. Structural and policy-driven educational contexts, including both physical and organizational characteristics of schools, shape student developmental outcomes (Abbasi and Luo, 2025). Within school organization, expansion or merger of schools alters students’ commuting times and distances, profoundly impacting academic performance (Tigre et al., 2017). The effect of commuting time on Students’ academic performance is also somewhat related to the commuting method used. However, researchers have differing opinions on the role of commuting method in the relationship between commuting time and student performance (García-Hermoso et al., 2017). Martínez-Gómez et al., 2010 found that Spanish students who chose active commuting modes exhibited higher cognitive ability scores. Nevertheless, the impact of transportation modes on students’ academic performance shows gender characteristics, with female students opting for active commuting demonstrating significantly better academic performance and cognitive abilities compared to their male counterparts (Jamil et al., 2022). Ruiz-Hermosa et al. (2019) conducted a systematic analysis of 12 studies and found no significant association between active commuting and students’ cognitive performance and academic performance.

Prolonged commuting time leads to psychological health issues for students and reduces their learning engagement. A substantial body of literature indicates a significant correlation between commuting time and adult mental health (Nie and Sousa-Poza, 2018; Friman et al., 2017; Wu, 2017). Ferreira Lima et al. found that when students’ commuting time exceeds a certain range, it can trigger negative psychological responses such as stress, anxiety, and depression (Lima et al., 2024). Additionally, Nigel Page et al. confirmed that longer commuting times and distances are linked to lower student satisfaction and decreased frequency of academic engagement (Page et al., 2021). Positive psychological well-being and resilience are essential resources for students to overcome academic difficulties and obstacles. Whether commuting time can impede students’ academic performance by triggering mental health issues in students—including burnout, anxiety, and stress—has not been thoroughly examined in existing research. Moreover, increased commuting time can distract students, diminish their interest in school activities, reduce their educational engagement and investment in learning. Ultimately, this can lead to a decline in academic performance, low morale, increased dropout rates (Bammou et al., 2024).

The Conservation of Resources (COR) theory and Commuting Stress Theory (CST) are often used to explain the impact of commuting time on adult work performance, innovative behavior, well-being, and satisfaction (Chatterjee et al., 2019; Wiese et al., 2024). The COR theory posits that the better the commuting control experience, the fewer resources are lost during the commuting process. As a result, individuals have more resources and motivation to engage in behaviors that contribute to work performance (Gan et al., 2024). CST posits that excessive commuting time not only increases objective and subjective stress but also has a squeezing effect on personal discretionary time, such as time for exercise and social leisure, thereby hindering individual job opportunities, resource acquisition, and work and academic performance (Sun and Wu, 2019; Pengfei and Xiang, 2018; Balabanian 2020). However, COR and CST theories have rarely been used to explain the intrinsic mechanisms by which commuting time affects students’ educational outcomes (academic performance and cognitive abilities). This study attempts to introduce COR and CST theories to analyze the impact of commuting time on academic performance, thereby deepening the applicability of both theories.

Through a review of existing studies, the following findings emerge: First, researchers have only examined the impact of commuting time on students’ academic performance without analyzing whether this impact exhibits certain group differences. Additionally, there has been insufficient in-depth analysis on whether commuting time affects students’ academic performance through learning engagement and psychological burnout. This study attempts to analyze the mediating effects of learning engagement and psychological burnout between commuting time and students’ academic performance, addressing the previous research neglect of these factors. Second, commuting time is influenced by students’ commuting method; however, there is no consistent conclusion on whether a student’s commuting method moderates the intensity of the effect of commuting time on students’ academic performance. This study seeks to explore whether commuting mode significantly moderates the effects of commuting time on students’ academic performance. Third, previous studies have utilized small sample sizes to analyze the effects of commuting time on students’ academic performance, which limits the generalizability of the findings. Therefore, this study uses the 2014–2015 China Education Panel Survey (CEPS) data to address the shortcomings of previous research in terms of sample representativeness.

This study aims to explore the impact of commuting time on the students’ academic performance and the underlying mechanisms. Specifically, the research objectives are: (1) to analyze the effects of commuting time on the students’ academic performance; (2) to examine the differences between various groups; (3) to investigate the mediating effects of psychological burnout and learning engagement in the relationship between commuting time and students’ academic performance; and (4) to assess the moderating role of commuting method in the relationship between commuting time and students’ academic performance.

To investigate the aforementioned issues, this study utilizes the 2014–2015 China Education Panel Survey (CEPS) follow-up data, which possesses high representativeness and unique value. Firstly, the 2014–2015 CEPS follow-up data was collected through a sampling survey method, drawing samples from 28 counties, 112 schools, and 438 classes across the country. The survey encompasses various aspects of students’ development experiences, physical and mental health, learning engagement, family economics, parental education levels, extracurricular activities, teacher-student relationships, social behavior development, educational expectations, and more, spanning multiple areas of school and family education, resulting in a total of 10,750 valid samples. Secondly, the 2014–2015 CEPS follow-up data includes newly added items related to students’ “commuting time” and “commuting method” based on the 2013–2014 CEPS baseline survey. This is currently the only publicly available nationwide survey data concerning student commuting time and commuting modes, providing solid data support for this research.

The remaining contents of this study are as follows: a review of relevant literature, the presentation of research hypotheses and theoretical foundations; the explanation of research design; the display of empirical analysis results; a report on research findings; and a statement regarding research limitations and future directions for exploration.

### 1.1 The influence of commuting time on students’ academic performance

Commuting time refers to the duration students spend traveling from home to school or from their residence to school. In the environment where students are situated, the convenience of transportation is a significant factor in shaping students’ academic performancet, and reducing the stress associated with commuting is crucial for improving academic performance (Hopson et al., 2024). Som Pal Baliyan surveyed 168 students and found that those with longer commutes scored 9.717 points lower in mathematics than those with shorter commutes, but commuting had no significant effect on English scores (Pal Baliyan and Khama, 2020). Robson Tigre used a randomized assignment design to estimate the impact of commuting time on standardized math scores among primary school students in Recife, Brazil. The study found that for every minute of increased commuting time, students’ math scores decreased by 0.166 points (*p* < 0.001). While the effect of commuting time was consistent across different score ranges, it had a more pronounced impact on high-scoring students than on low-scoring ones (Tigre et al., 2017). Olalekan discovered a significant positive correlation between commuting time and math scores (*r* = 0.054, *p* < 0.001). commuting time not only hinders academic improvement but also affects the continuity and persistence of students’ participation in learning tasks (Idowu, 2016). Kristin Blagg evaluated the commuting times of kindergarten, sixth grade, and ninth grade students in Washington D.C. found that the longer the commute, the higher the rates of student transfers, dropouts, and absences. However, the impact of students’ commuting time on their standardized academic performance varies across different subjects. Students with longer commuting times tend to perform better in English reading and mathematics compared to those with shorter commuting times (Blagg, 2018). The commuting time not only affects the physical distance but also the associated time distance, both of which can influence students’ academic performance. Research has shown that the time distance and physical distance to school have a significant positive impact on students’ academic performance. Compared to physical distance, time distance has a more pronounced effect on academic performance (Zhao and Wu, 2011). Emerson D. Peteros found that the higher the accessibility to the school, the better the students’ academic performance (Peteros et al., 2022).

*H1*: Commuting time has a significant hindering effect on students ‘academic performance, that is, the longer the commuting time, the less conducive to students’ academic performance improvement.

### 1.2 The differential impact of commuting time on academic performance among students from different groups

The impact of commuting time on students’ academic performance varies, primarily due to individual and family characteristics. Regarding gender, commuting time has a more negative effect on male work performance than on female performance (He et al., 2021). In terms of age, considering the immaturity of social development and the lack of safety awareness among students, parents often choose nearby schools to enhance safety. In terms of family, commuting time has a greater negative impact on the academic performance of students from poor families. Sarah A. Cordes found that commuting time significantly negatively predicts reading scores for students from middle-and low-income families, with a decrease of 0.057 points for every minute of increased commuting time (Cordes et al., 2022). The educational level of parents also affects students’ commuting time. Students whose mothers have completed education below high school have higher average commuting times compared to those who have received college or undergraduate degrees (Fast, 2020). In terms of urban–rural disparities, cities possess more concentrated educational resources than rural areas, including highly educated teachers, ample educational funding, and high-impact academic activities. This results in urban students demonstrating significantly higher cognitive abilities than their rural counterparts (Abbasi et al., 2022). In terms of residential location, urban students experience a significant drop in scores by 0.069 units for every minute of increased commuting time, while rural students see a significant decline of 0.095 units (Ding and Feng, 2022). In summary, commuting time is more detrimental to the academic performance of rural students than to urban students. Regarding boarding and commuting, the distance to school has a greater negative impact on the academic performance of commuting students compared to boarding students (Jia, 2014). In the Chinese context, the commuting time and distance to school are embedded in family economics, leading to the phenomenon of “elite capture.” High-income families ensure their children receive quality educational resources by purchasing school district houses, thereby shortening the spatial and temporal distance between home and school, ensuring the convenience and safety of commuting. Families with better economic conditions typically reside near school districts, while low-income families often live in suburban or remote rural areas. The disparity in distance between home and school results in varying levels of commuting stress for students, which significantly impacts their academic development. Parents hope their children will excel. Parents’ educational expectations for their children influence educational investments. Differences in parents’ education levels, occupations, and income affect their ability to compete for school district housing and quality educational resources. Families with substantial economic and cultural capital are more inclined to invest in high-quality school districts, considering their children’s future educational needs and the returns from good employment opportunities (Jiang, et al., 2022). Liu and Xie (2018) analysis of the relationship between neighborhood effects and students’ academic performance found that middle-class families can leverage their cultural and economic advantages by purchasing school district housing, which helps their children gain priority access to regional educational resources and mitigate external factors that could negatively impact their development, such as poor community environments and long commutes.

*H2*: The impact of commuting time on students’ academic performance is heterogeneous, that is, the impact of commuting time on students’ academic performance shows significant differences due to different individual characteristics and family factors of students.

### 1.3 The mediating role of learning investment

The reason why commuting time can hinder students’ academic development is that it is competitive, reducing students’ study time and thus impeding their academic progress. Longer commuting times mean students have to spend more time on public transportation or other means of getting to school. Even with parental or school bus pick-ups, this reduces students ‘after-school study time, which in turn affects their grades, sleep, and participation in extracurricular activities. Sarah A. Cordes analyzed the relationship between commuting and academic performance using data from 120,000 third to sixth-grade students in New York City during the 2011–2017 period. The analysis found that students who took long-distance buses or ultra-long buses to school had attendance rates that decreased by 17 and 27.9%, respectively, and their long-term absenteeism rate increased by 1 percentage point. Attendance was significantly positively correlated with reading and math scores (Cordes et al., 2022). When families are too far from schools, students often need to wake up early to get to school on time, which can lead to insufficient sleep, affecting their concentration and cognitive activity, and hindering their engagement in learning. Finley Edwards analyzed the impact of daily school start times on academic performance using data from all middle school students in Wake County, North Carolina, from 1999 to 2006. The study found that earlier school start times reduced students’ sleep time, and insufficient sleep hindered cognitive development and learning engagement, ultimately leading to lower academic performance (Edwards, 2012). Marc Frenette analyzed the impact of the distance between high school residences and universities on college choices and attendance rates. The study found that the increased spatial distance between high schools and universities significantly reduced enrollment rates. High school students from low-income families who live outside the commuting range (more than 80 miles from their university) have a 37% lower enrollment rate compared to those living near their universities (Frenette, 2004). Geographically, long commuting distances and times can impose significant emotional and psychological burdens on students, hindering their ability to choose and engage in time-demanding courses, which over time can impede academic performance (Kobus et al., 2015). Long commuting times also expose students to more uncontrollable situations, such as traffic congestion, delayed bus services, and adverse weather conditions, all of which can affect students’ punctuality. The longer the commuting time and distance, the higher the likelihood of students being late due to traffic delays. Charis Loong found that weather and commuting time significantly impact students’ punctuality and learning state (Loong et al., 2017). In terms of weather, students experience higher learning efficiency in warm and sunny weather compared to cold and rainy weather, with the former being 6.07 times more effective than the latter. Regarding punctuality, students are 95% less likely to be late when commuting in warm and dry weather compared to cold and snowy weather. Traffic and weather conditions can affect students’ punctuality and sense of time urgency. Werner found a significant causal relationship between student commuting and punctuality, with students who commute by bicycle or on foot being more likely to be late compared to those who commute by private car or train (Werner et al., 2015).

*H3*: Learning investment functions as a mediating variable between commuting time and academic performance. Specifically, increased commuting time restricts students’ investment in learning, which consequently impedes their academic performance.

### 1.4 The mediating role of psychological fatigue

The time spent commuting has a detrimental effect on students’ mental health, particularly the prolonged commute can lead to psychological fatigue and burnout. According to the stress–stress theory, continuous stress and workload can trigger stress responses in individuals, leading to anxiety, tension, and fatigue, which can harm mental health (Miao and Wang, 2021). Students spend a significant amount of time traveling between school and home each day, which can increase their travel pressure and negative emotions, leading to psychological burnout. Psychological burnout is an important indicator of students’ enthusiasm for learning and their sense of efficacy. If students experience burnout due to long commutes, they may lose focus in class, have reduced interest in learning, and consequently see a decline in academic performance. From the perspective of resource consumption theory, extended commuting times increase the exposure of students to crowded public transportation, such as buses, subways, and coaches. To arrive at school on time, students often sacrifice sleep, and long-term sleep deprivation not only leads to physical fatigue but also reduces their sense of self-efficacy in learning. Research indicates that for every additional unit of commuting time, the frequency of mental depression, mental tension, restlessness, and difficulty in doing things increases by 0.0026, 0.0022, 0.0019, and 0.0023 units, respectively (Sun and Wu, 2019). The longer the commute, the more sensitive students’ nerves become to road congestion and traffic noise, and the psychological pressure they endure intensifies over time, ultimately damaging their psychological resilience. Ding analyzed the impact of commuting on mental health and academic performance of primary and middle school students. The study found that the longer the commuting time, the more likely it is to lead to negative and pessimistic emotions in students. The low mood of students increases the frequency of absence and leave, which will reduce students’ academic performance in the short term and damage their long-term educational results (Ding and Li, 2023).

*H4*: Psychological burnout mediates the relationship between commuting time and students’ academic performance, that is, commuting time will deepen students’ psychological burnout, which in turn hinders their academic performance development.

### 1.5 The regulating effect of commuting method

Commuting time is influenced not only by the distance but also by the choice of commuting methods. According to Easton and Ferrari, students’ commuting method are categorized into active and passive commuting based on their mode of transportation (Easton and Ferrari, 2015). Passive commuting involves students using family cars, buses, taxis, trains, or subways to get to school; whereas active commuting includes students riding bicycles, skateboards, or walking to school. Hinckson et al.,(2014) found that students who commute actively benefit from the convenience of motorized vehicles, resulting in shorter commuting times. However, this can lead to increased sedentary time at school, potentially causing obesity and myopia, which can negatively impact academic performance. Compared to passive commuting, active commuting provides more opportunities for physical exercise (Marique et al., 2013). A study by Palma found that active commuting increases the intensity of physical activity, which is beneficial for improving cardiorespiratory health and cognitive development (Chillon et al., 2010). Passive commuting reduces students’ perceived well-being. Perceived well-being refers to a positive psychological state, including evaluations of daily life, mental health, and physical health, whether positive or negative (Clark et al., 2020).

*H5*: The effect of commuting method adjustment on students’ academic performance. In short, active commuting can alleviate the blocking effect of commuting time on academic performance, while passive commuting will aggravate the negative effect of commuting time on academic performance.

### 1.6 Analysis model

The Commuting Stress Theory (CST) and Conservation of Resources Theory (COR) are classic frameworks that explain how commuting duration influences work performance and psychological experiences. CST posits that commuting stress refers to an individual’s psychological or physiological responses triggered by adverse outcomes during the commute, and it often encompasses subjective evaluations of the commuting behavior itself (Demiral, 2018). Extended commutes are associated with a range of negative consequences, including emotional exhaustion, mental fatigue, reduced well-being, and diminished work performance (Chen et al, 2022). From the CST perspective, excessive commuting demands induce psychological strain and stress in individuals. In particular, when lacking support from work-related resources, persistent psychological strain and stress erode individual resilience, leading to burnout and subsequent declines in performance output. Additionally, CST emphasizes the physical and psychological burdens imposed on commuters by uncontrollable factors, such as public transit malfunctions, traffic congestion, and unexpected incidents. These uncontrollable factors elicit stress responses because of the transactional “stress-coping” relationship between individuals and external stressors (Sumicad et al., 2024). Longer commutes increase the likelihood of encountering such uncontrollable factors. When individuals perceive that these factors will hinder their ability to achieve work goals and fulfill responsibilities, and they lack the coping skills or confidence to address them, feelings of anxiety, tension, and worry begin to emerge.

**Figure 1:**
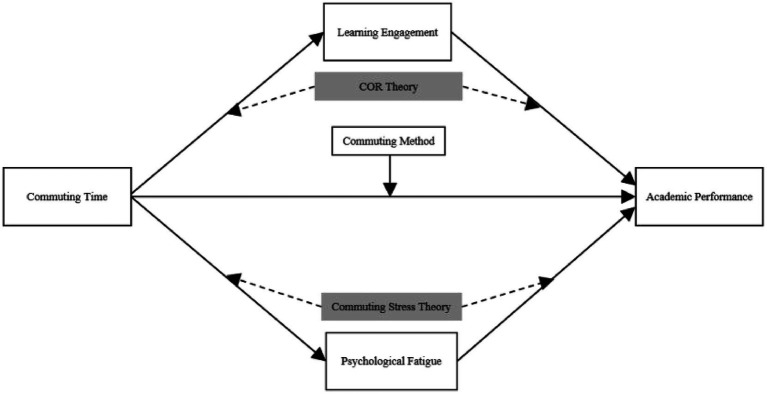
Research theoretical model.

The Conservation of Resources Theory (COR) considers all factors that facilitate the achievement of work performance as resources, while those that impede goal attainment are viewed as demands (Li et al., 2025). For students, commuting constitutes a demand that depletes their personal resources needed to reach school, particularly time and energy. The Conservation of Resources Theory (COR) posits that individuals tend to maintain, protect, and cultivate the resources they deem important. When resources are at risk of loss, individuals take preventive measures to mitigate such losses. Extended commutes consume the limited cognitive and emotional resources of individuals, thereby reducing the resources available for work. Those with limited resources are more likely to experience stress responses aimed at preventing further resource depletion when faced with high pressure and demanding tasks (Hobfoll et al., 2018). COR focuses more on how commuting duration affects individuals’ emotional responses. Lengthy commutes can lead to travel stress, boredom, and frustration, especially when commuting is complicated by traffic congestion or a lack of safety, factors that can easily trigger negative emotions in commuters. Students who commute long distances often feel tired or even exhausted due to factors such as lengthy travel times, noise disturbances, and adverse weather conditions. The depletion of subjective psychological resources compels students to conserve their limited remaining resources, resulting in insufficient energy for learning. Based on the aforementioned research hypotheses and related theories, the analytical model constructed for this study is illustrated in Figure 1.

## 2 Methods

### 2.1 Data sources

This study draws data from the 2014–2015 China Education Panel Survey (CEPS) follow-up data, which surveyed eighth-grade students and yielded a total of 10,750 valid samples. As the 2014–2015 CEPS follow-up data builds upon the 2013–2014 CEPS baseline survey, it introduces new items regarding students’ commuting time and modes of transportation while removing personal and household information. Therefore, this study matched the 2013–2014 CEPS baseline data with the 2014–2015 CEPS follow-up data using student ID numbers to supplement individual background and household information, ultimately yielding 9,449 valid samples. All these samples successfully completed follow-up tracking, but data gaps existed in certain items, meaning not all variables possessed complete data across all 9,449 samples.

### 2.2 Variables and measurements

#### 2.2.1 Commuting time is the predictor variable

The predicted variable is commuting time, quantified through the questionnaire question “How long does it take you to commute from home to school?” and classified as a continuous variable. Commuting time is measured in minutes.

#### 2.2.2 Academic performance is the outcome variable

The outcome variable represents the academic performance of students. Raw test scores in Chinese, mathematics, and English serve as indicators of academic performance.

#### 2.2.3 Psychological fatigue and learning engagement serve as mediating variables

Psychological burnout is measured using the questionnaire item “lacking motivation to do things”. This variable is continuous, ranging from 1 (“never”) to 5 (“often”). Academic research defines learning engagement as comprising three dimensions: cognitive engagement, behavioral engagement, and affective engagement. However, the questionnaire lacks specific survey items measuring students’ cognitive, behavioral, and affective engagement. Therefore, this study uses the item “feeling so depressed that I cannot concentrate on tasks”as a proxy variable for students’ learning engagement, employing reverse scoring to reflect behavioral tendencies related to learning engagement. Learning engagement is a continuous variable, with 1 indicating “never” and 5 indicating “often.”

#### 2.2.4 Commuting method is a moderator variable

Commuting methods are categorized into active and passive commuting. Active commuting includes cycling and walking, while passive commuting encompasses other transportation methods such as buses, subways, private cars, electric vehicles, and boats. Commuting mode is a categorical variable. A value of 1 indicates active commuting, while 0 indicates passive commuting.

#### 2.2.5 Individual characteristics and family environment are control variables

Personal traits and household conditions may influence academic performance. This study controls for students’ personal attributes (including gender, age, grade level, only-child status, health status, and boarding status) and family characteristics (including family structure, parental education levels, parental relationship quality, and household economic status). Since the 2014–2015 China Education Tracker Survey data were collected in the fall of 2014 and spring of 2015, this study incorporates time as a fixed effect in the analysis. Specific variable settings and observation indicators are detailed in Table 1.

**Table 1:** Variable settings and descriptions.

Variable category	Variable name	Measurement indicator	Measurement method	N	Mean/Percentage
Dependent variable	Academic performance	Chinese language score	Raw score	9,328	81.36
		Mathematics score	Raw score	9,330	75.36
		English score	Raw score	9,320	72.91
Independent variable	Commuting time	Commuting time from home to school	Specific duration	9,097	24.34
Mediating variable	Learning engagement	Concentration on Tasks	1 = Never; 5 = Always	9,449	3.50
	Psychological fatigue	Lacking motivation to work	1 = Never; 5 = Always	9,399	2.14
Moderator variable	Commuting method	Passive commuting	0 = Passive Commuting	4,482	47.1
		Active commuting	1 = Active Commuting	4,967	52.9
Control variables	Gender	Female	0 = Female	4,592	48.6
		Male	1 = Male	4,857	51.4
	Age	Date of birth	2015-Date of Birth	9,279	15.36
	Only child	No	0 = No	4,446	47.1
		Yes	1 = Yes	5,003	52.9
	Health status	Level of overall health	1 = Very poor; 5 = Very good	9,340	4.10
	Boarding	No	0 = No	6,649	70.4
		Yes	1 = Yes	2,800	29.6
	Household Registration	Urban	0 = Urban	4,606	48.7
		Rural	1 = Rural	4,843	51.3
	Father’s education level	Father’s education level	1 = No education 9 = Graduate degree or higher	9,247	4.32
	Mother’s education level	Mother’s education level	1 = No education; 9 = Graduate degree or higher	9,278	4.02
	Parental relationship	Parental relationship	0 = Poor	7,965	84.3
			1 = Very good	1,484	15.7
	Family economy	Family economic status	1 = Extremely difficult; 5 = Very wealthy	9,423	2.91

### 2.3 Research methods

First, this study employs linear regression analysis to estimate the effect of commuting time on student academic performance, controlling for individual characteristics, family factors, and the survey implementation period. Second, to test the robustness of the impact of commuting time on academic performance, the study uses a substitution approach by replacing students’ scores in Chinese, math, and English with their raw cognitive ability test scores, as well as their scores on learning difficulties in Chinese, math, and English to examine the impact of commuting time on students’ academic performance. If the regression results are significant, it indicates that the research conclusions are robust. Finally, the PROCESS procedure was used to analyze the mediating effects of learning engagement and psychological exhaustion, while interaction terms were employed to examine the moderating effect of commuting mode.

## 3 Results

### 3.1 Key variable correlation analysis

Using Prism software, the study analyzed the correlations among key variables, with results shown in Table 2. Table 2 indicates that commuting time exhibits significant negative correlations with students’ Chinese scores (*r* = −0.047, *p* = 0.000), mathematics scores (*r* = −0.053, *p* = 0.000), English scores (*r* = −0.087, *p* = 0.000), and academic engagement (*r* = −0.084, *p* = 0.000). Commuting time exhibits a significant negative relationship with academic engagement (*r* = −0.084, *p* = 0.000) and a significant positive relationship with psychological fatigue (*r* = 0.079, *p* = 0.000). Students’ learning engagement showed significant positive correlations with Chinese language scores (*r* = 0.051, *p* = 0.000), mathematics scores (*r* = 0.095, *p* = 0.000), and English language scores (*r* = 0.102, *p* = 0.000). Psychological fatigue exhibited significant negative correlations with Chinese language scores (*r* = −0.029, *p* = 0.000), mathematics scores (*r* = −0.061, *p* = 0.000), and English language scores (*r* = −0.075, *p* = 0.000).

**Table 2:** Correlation analysis results.

Variables	commuting time	Chinese score	Math score	English score	Learning engagement	Mental fatigue	Commuting method
Commuting time	1	−0.047**	−0.053**	−0.087**	−0.084**	0.079**	−0.148**
Chinese language scores		1	0.679**	0.694**	0.051**	−0.029**	−0.078**
Mathematics scores			1	0.743**	0.095**	−0.061**	−0.079**
English language scores				1	0.102**	−0.075**	−0.079**
Learning engagement					1	−0.642**	0.019
Psychological fatigue						1	−0.021*
Commuting method							1

*p < 0.05; **p < 0.01; ***p < 0.001.

### 3.2 Analysis of the impact of commuting time on academic performance

Table 3 presents the results of the linear regression model. Model 1 demonstrates the impact of commuting time on students’ overall academic performance after controlling for individual student characteristics, family factors, and time-fixed effects. Overall academic performance is calculated by averaging the scores in Chinese, mathematics, and English. According to Model 1, a one-minute increase in commuting time leads to a 0.045-point decrease in students’ overall academic performance, which is statistically significant at the 0.1% level. Models 2 through 4 provide estimates of the effects of commuting time on students’ Chinese, mathematics, and English scores. Models 2, 3, and 4 show that a one-minute increase in commuting time results in a 0.04-point decrease in Chinese scores, a 0.032-point decrease in mathematics scores, and a 0.05-point decrease in English scores. These changes are statistically significant at the 1, 1, and 0.1% significance levels. This indicates that commuting time impedes students’ academic performance and undermines their academic development, thereby supporting research hypothesis H1.

**Table 3:** Baseline regression model results.

Dependent variable		Overall score (M1)		Chinese score (M2)		Mathematics score (M3)		English score (M4)	
		Beta	*P*	Beta	*P*	Beta	*P*	Beta	*P*
Independent variable	Commuting time	−0.045*** (0.008)	0.000	−0.040** (0.007)	0.000	−0.032** (0.011)	0.005	−0.050*** (0.010)	0.000
Control variable	Male	0.007 (0.524)	0.519	0.012 (0.435)	0.260	0.001 (0.683)	0.915	0.007 (0.631)	0.532
	Age	0.009 (0.222)	0.417	−0.004 (0.185)	0.681	0.023* (0.290)	0.035	0.000 (0.268)	0.984
	Only child	0.027* (0.564)	0.020	0.005 (0.468)	0.643	0.027* (0.735)	0.021	0.035** (0.680)	0.003
	Health status	0.017 (0.280)	0.121	0.009 (0.232)	0.397	0.012 (0.365)	0.273	0.023* (0.337)	0.032
	Boarding	−0.013 (0.647)	0.298	0.021 (0.537)	0.091	−0.007 (0.843)	0.558	−0.037** (0.780)	0.002
	Rural household	−0.147*** (0.565)	0.000	−0.088*** (0.469)	0.000	−0.110*** (0.736)	0.000	−0.185*** (0.681)	0.000
	Father’s educational attainment	0.006 (0.177)	0.689	0.0264 (0.149)	0.095	0.000 (0.231)	0.976	−0.001 (0.214)	0.919
	Mother’s educational attainment	0.035* (0.183)	0.016	0.044** (0.152)	0.003	0.032* (0.239)	0.030	0.024 (0.221)	0.104
	Parental relationship	−0.007 (0.720)	0.490	−0.007 (0.598)	0.531	−0.007 (0.939)	0.532	−0.006 (0.868)	0.548
	Family economic status	0.014 (0.503)	0.195	0.020 (0.417)	0.074	0.014 (0.655)	0.222	0.008 (0.606)	0.441
	*N*	8,488		8,496		8,498		8,489	
	*R*-squared	0.031		0.015		0.019		0.050	
	Adj *R*-squared	0.030		0.013		0.018		0.048	
	*F*	22.776***		10.430***		13.741***		37.035***	
	Time-fixed effect	Yes		Yes		Yes		Yes	

*P < 0.05; **P < 0.01; ***p < 0.001. Coefficients are standardized coefficients (Beta), with standard errors (S.E.) in parentheses. Gender is referenced to “female”; only children are referenced to “non-only children”; boarding is referenced to “day students”; Family type is compared to “urban family”; Parental relationship is compared to “poor”.

Table 3 also indicates that certain individual and family characteristics significantly influence students’ academic performance. Specifically, in Model 1, the overall academic performance of only children is significantly higher than that of non-only children; students from rural families generally achieve lower academic performance than those from urban families; the higher the mother’s educational attainment, the better the overall academic performance of the students. In Models 2, 3, and 4, older students demonstrated superior mathematics performance; only children significantly outperformed non-only children in mathematics and English; better health status correlated with higher English scores; boarding arrangements negatively impacted English performance; students from rural households scored significantly lower in Chinese, mathematics, and English compared to their urban counterparts; and maternal education level also positively influenced students’ mathematics performance.

### 3.3 Endogeneity test

Due to potential omitted variable bias or self-selection bias in the sample, estimates of the effect of commuting time on academic performance among students may be biased. To address endogeneity, this study employs a Heckman two-stage model. The first stage utilizes a Probit model with “commuting status” as the dependent variable. Students with commuting times above the mean are coded as 1 (high commuting group), while those below the mean are coded as 0 (low commuting group). Simultaneously, factors influencing students’ commuting status (high/low) were introduced. These factors include gender, age, only child status, boarding status, health level, family type, parental education level, parental relationship, household economic status, and a dummy variable for the year. This approach allows for the estimation of the inverse Mills ratio (IMR), which is then introduced as a control variable into the Heckman results model. The results are presented in Table 3. As shown in Models M1, M2, M3, and M4 of Table 3, the regression coefficients for the IMR are all statistically significant. This indicates that the first-stage Heckman selection model is appropriate and effectively controls for sample self-selection bias. Furthermore, Table 4 demonstrates that commuting time exerts a significant heterogeneous effect on overall academic performance, Chinese language scores, mathematics scores, and English language scores among students. This confirms that the conclusion supporting research hypothesis H1 is robust.

**Table 4:** Regression results of the Heckman two-stage model.

Dependent variable	Overall score (M1)		Chinese score (M2)		Mathematics score (M3)		English score (M4)	
	Beta	*P*	Beta	*P*	Beta	*P*	Beta	*P*
Commuting time	−0.023** (0.009)	0.010	−0.022** (0.008)	0.004	−0.017* (0.012)	0.012	−0.032** (0.011)	0.005
IMR	−0.881* (0.394)	0.025	−0.119* (0.327)	0.038	1.380** (0.513)	0.007	1.122* (0.475)	0.018
Control variable	Yes		Yes		Yes		Yes	
Time fixed effect	Yes		Yes		Yes		Yes	
*N*	8,909		8,906		8,911		8,901	
*R*-squared	0.030		0.013		0.020		0.047	
Adj *R*-squared	0.028		0.011		0.018		0.046	
*F*	25.05***		10.72***		16.11***		40.41***	

*P < 0.05; **P < 0.01; ***P < 0.001; values in parentheses indicate standard error (S.E.).

### 3.4 Heterogeneity analysis of the effect of commuting time on academic performance

Table 5 presents the analysis of the heterogeneity in students ‘individual and family characteristics. Regarding gender, commuting time has a more significant impact on female students’ academic performance. For every minute of increased commuting time, female students’ scores drop by 0.092 points, while male students’ scores decrease by 0.053 points. Rahma M. Msoffe found that the negative impact of commuting time on female students’ academic performance is more pronounced. This is because male students, after a period of commuting, are generally more energetic and mentally alert compared to female students. Additionally, female students are more sensitive to the stress caused by commuting, leading to lower course participation, higher dropout rates, and more frequent tardiness, ultimately resulting in a greater decline in their academic performance (Rahma M. Msoffe, 2023).

**Table 5:** Results of heterogeneity analysis.

Variable	Female	Male	Only child	Non-only child	Urban	Rural	Resident student	Non-resident student	Low-income family	High-income family
	(M1)	(M2)	(M3)	(M4)	(M5)	(M6)	(M7)	(M8)	(M9)	(M10)
Commuting time	−0.091***(0.012)	−0.053***(0.011)	−0.078***(0.011)	−0.061***(0.011)	−0.084***(0.014)	−0.032* (0.010)	−0.050**(0.010)	−0.059***(0.014)	−0.019 (0.023)	−0.080*** (0.008)
Time fixed effect	Yes	Yes	Yes	Yes	Yes	Yes	Yes	Yes	Yes	Yes
*N*	4,348	4,626	4,750	4,224	4,380	4,594	2,676	6,298	1,321	7,652
*R*-squared	0.009	0.003	0.010	0.006	0.008	0.001	0.002	0.004	0.005	0.007
Adj *R*-squared	0.008	0.002	0.009	0.006	0.008	0.001	0.002	0.004	0.003	0.007
*F*	18.958***	6.574***	26.613***	13.101***	18.111***	2.389***	3.311***	13.256***	3.226***	26.631***

*P < 0.05; **P < 0.01; ***P < 0.001; values in parentheses indicate standard error (S.E.).

Regarding only children, the impact of commuting time on their academic performance is more pronounced than that on non-only children. For every minute of increased commuting time, only children’s academic performance drops by 0.78 points, while non-only children’s performance decreases by 0.061 points. The family structure of only children is generally more homogeneous compared to non-only children, and the stress and fatigue experienced by only children due to commuting time are less likely to be alleviated through peer communication, thus exacerbating the negative effects of commuting time on their academic performance.

Regarding household registration, commuting time has a more significant negative impact on the academic performance of urban students compared to rural students. For every minute that commuting time increases, urban students’ scores drop by 0.082 points, while rural students’ scores decrease by only 0.032 points, with the former being statistically significant at the 0.1% level. The reason for this is that although urban areas offer a variety of transportation options, students in cities often face traffic congestion during their commutes, leading to longer commuting times compared to rural students. Therefore, the negative impact of commuting time on urban students’ academic performance is more pronounced. In contrast, middle school students in rural areas typically prefer boarding schools, which significantly reduces their commuting time, making it easier for them to focus on their studies and mitigate the negative effects of commuting time on their academic performance.

Regarding the academic performance gap between residential and non-residential students, commuting time exerts a markedly different impact. Data reveals that for every additional minute of commuting time, non-residential students’ scores decrease by 0.059 points, while residential students’ scores decline by only 0.05 points. Residential students live on campus weekly, requiring travel only during holidays or weekends, resulting in significantly shorter commuting times compared to non-residential students. Non-residential students travel daily between home and school, facing longer distances, extreme weather, and unexpected disruptions. These factors not only consume more energy but also reduce time for interaction with peers and teachers, hindering their participation in higher-order learning activities. Consequently, commuting time exerts a more pronounced negative impact on the academic performance of non-residential students.

Regarding family economic conditions, commuting time has no significant impact on the academic performance of students from economically disadvantaged families. However, it significantly negatively affects the academic performance of students from families with average or affluent economic backgrounds. Families with strong financial resources tend to provide their children with more diverse educational opportunities, particularly in extracurricular activities. This often results in students from better-off families having longer and more frequent commutes, thus experiencing a greater negative impact from commuting time.

Heterogeneity tests indicate that the negative impact of commuting time on student academic performance varies significantly, primarily attributable to individual differences among students and variations in family factors. Therefore, the findings support Hypothesis H2.

### 3.5 Robustness analysis of the effect of commuting time on academic performance

This study examined the robustness of commuting time’s impact on academic performance among students by replacing the dependent variable. Raw scores from cognitive ability tests and difficulty ratings for Chinese, mathematics, and foreign language learning (1 = not difficult at all, 4 = extremely difficult) were used as dependent variables. Detailed regression analysis results are presented in Table 6.

**Table 6:** Durability test results.

Dependent variable	Cognitive ability (M1)		Struggling with Chinese language studies (M2)		Difficulty with mathematics learning (M3)		Struggling with foreign language learning (M4)	
	Beta	*P*	Beta	*P*	Beta	*P*	Beta	*P*
Commuting time	−0.063*** (0.002)	0.000	0.049*** (0.001)	0.000	0.040*** (0.001)	0.000	0.063*** (0.003)	0.000
Control variable	Yes		Yes		Yes		Yes	
Time fixed effect	Yes		Yes		Yes		Yes	
*N*	8,600		8,578		8,581		8,579	
*R*-squared	0.033		0.028		0.039		0.067	
Adj *R*-squared	0.031		0.026		0.037		0.065	
*F*	24.113***		20.327***		28.844***		51.032***	

*P < 0.05; **P < 0.01; ***P < 0.001; values in parentheses indicate standard error (S.E.).

As shown in Table 6, commuting time exerts a significant negative impact on students’ cognitive abilities. For every additional minute of commuting time, students’ scores on cognitive ability tests decrease by 0.063 points. Extended commutes may induce anxiety, tension, and fatigue, causing students to experience mental sluggishness in class, which has a detrimental effect on cognitive development. Regarding learning difficulty, commuting time increases the challenge of studying Chinese, mathematics, and foreign languages, making the learning process more arduous for students. The difficulty levels rise by 0.049, 0.040, and 0.063 units, respectively. Robustness tests reveal that even after replacing the dependent variable, commuting time continues to exert a significant negative impact on students’ academic performance.

### 3.6 Mediation effect analysis of learning investment and psychological fatigue

This study employed the Process procedure in SPSS 20 to conduct 2000 simulations on the research sample, analyzing the mediating effects of learning investment and psychological fatigue on the relationship between commuting time and academic performance among students. The specific results are presented in Table 6.

Regarding learning engagement, the estimated effects of commuting time on students’ Chinese scores (CT → LE → CS), math scores (CT → LE → MS), and English scores (CT → LE → ES) through learning engagement were −0.0021, −0.0046, and −0.0051, respectively. The corresponding 95% confidence intervals did not include zero, indicating that commuting time impedes learning engagement and consequently exerts a significant negative impact on students’ academic performance. This result supports research hypothesis H3. This study found that when the dependent variables were Chinese language scores, mathematics scores, and English language scores, the estimated effects of commuting time on students’ learning commitment were −0.003 (*p* = 0.000 < 0.001), −0.0026 (*p* = 0.000 < 0.001), and −0.0026 (*p* = 0.000 < 0.001), respectively, all significant at the 0.1% level. This indicates that longer commuting times correlate with lower learning commitment among students. Estimated values of learning engagement on Chinese language scores (LE → CS), mathematics scores (LE → MS), and English scores (LE → ES) were 0.4986 (*p* = 0.011 < 0.05), 1.7857 (*p* = 0.000 < 0.001), and 1.9809 (*p* = 0.000 < 0.001), respectively. This indicates that higher learning engagement among students correlates with more significant academic performance development.

Learning engagement serves as a critical supporting factor influencing students’ academic performance, significantly enhancing learning outcomes. As shown in Table 7, commuting time exerts a significant inhibitory effect on learning engagement. According to resource conservation theory, prolonged commuting drastically reduces students’ discretionary time, forcing them to cut back on leisure activities, physical exercise, social engagement, and advanced course selection (Hinckson et al., 2014). This time compression hinders students from building beneficial peer networks, fostering teacher-student interactions, and developing group belonging, thereby diminishing their interest and initiative in learning, ultimately impeding academic progress. While commuting provides a transitional buffer period between academic and social environments: enabling psychological and social adaptation through activities like listening to music, independent thinking, or reading: it also exposes students to adverse factors such as traffic noise, environmental pollution, and extreme weather, potentially disrupting mental relaxation. If commuting is viewed as a cost within the learning process, longer commutes consume more psychological resources, increasing the burden of completing academic tasks. To conserve resources, students may divert limited mental energy away from learning and teacher-student interactions, leading to avoidance behaviors. Sustained engagement in learning becomes difficult to maintain, ultimately resulting in declining academic performance.

**Table 7:** Test results for the mediating effect of learning investment on psychological burnout.

Independent variable	Mediating variable	Dependent variables	Path	Effect size	S.E	*P*	95%LLCI	95%LLUI
Commuting time	Learning engagement (LE)	Chinese score (CS)	CT → CS	−0.046***	0.0065	0.000	−0.0412	−0.0015
			CT → LE	−0.003***	0.0004	0.000	−0.0033	−0.0019
			LE → CS	0.499*	0.1965	0.011	0.1134	0.8838
			CT → LE → CS	−0.0020	0.0009	-	−0.0038	−0.0005
		Mathematics score (MS)	CT → MS	−0.0474***	0.0103	0.000	−0.0676	−0.0272
			CT → LE	−0.0026***	0.0004	0.000	−0.0033	−0.0019
			LE → MS	1.7857***	0.3098	0.000	1.1784	2.3929
			CT → LE → MS	−0.0046	0.0011	-	−0.0070	−0.0026
		English score (ES)	CT → ES	−0.0758***	0.0096	0.000	−0.0947	−0.0569
			CT → LE	−0.0026***	0.0004	0.000	−0.0033	−0.0019
			LE → ES	1.9809***	0.2896	0.000	1.4133	2.5485
			CT → LE → ES	−0.0051	0.0011	-	−0.0074	−0.0032
	Psychological fatigue (PF)	Chinese score (CS)	CT → CS	−0.0284***	0.0065	0.000	−0.0412	−0.0155
			CT → PF	0.0026***	0.0004	0.000	0.0019	0.0033
			PF → CS	−0.4986*	0.1965	0.0112	−0.8838	−0.1134
			CT → PF → CS	−0.0013***	0.0006	-	−0.0024	−0.0002
		Mathematics score (MS)	CT → MS	−0.0474***	0.0103	0.000	−0.0676	−0.0272
			CT → PF	0.0026***	0.0004	0.000	0.0019	0.0033
			PF → MS	−1.7857***	0.3098	0.000	−2.3929	−1.1784
			CT → PF → MS	−0.0046***	0.0011	-	−0.0069	−0.0027
		English score (ES)	CT → ES	−0.0758***	0.0096	0.000	−0.0947	−0.0569
			CT → PF	0.0026***	0.0004	0.000	2.0439	2.0996
			PF → ES	−1.9809***	0.2896	0.000	−2.5485	−1.4133
			CT → PF → ES	−0.0051	0.0011	-	−0.0075	−0.0032

*P < 0.05; **P < 0.01; ***P < 0.001; values in parentheses indicate standard error (S.E.).

Regarding psychological fatigue, the estimated effects of commute duration on students’ Chinese scores (CT → PF → CS), math scores (CT → PF → MS), and English scores (CT → PF → ES) through psychological fatigue were −0.0013, −0.0046, and −0.0051, respectively. The corresponding 95% confidence intervals for all three estimates did not include zero, indicating that psychological fatigue significantly mediates the relationship between commuting time and academic performance among students. Furthermore, longer commuting times correlate with greater psychological fatigue among students. Psychological fatigue significantly inhibits Chinese language performance (*α* = −0.4986, *p* = 0.0112 < 0.05), mathematics performance (*α* = −1.7857, *p* = 0.000 < 0.001), English scores (*α* = −1.9809, *p* = 0.000 < 0.001). This indicates that commuting time not only directly impacts academic performance but also indirectly influences it through psychological fatigue. In other words, psychological fatigue partially mediates the relationship between commuting time and students’ performance in Chinese, mathematics, and English, validating Hypothesis H4. From the perspective of commuting stress theory, an ideal commute implies that students should complete the journey from home or residence to school in the shortest possible time. However, students lack control over the efficiency of commuting vehicles or the convenience of commuting routes. This may lead to significant commuting stress during the journey. Once this commuting stress exceeds the psychological tolerance of students, it can trigger psychological reactions such as anxiety, anger, and dissatisfaction (Liu, 2024). For students with weak time management skills or a tendency toward procrastination, excessively long commutes impose heavy psychological burdens, making punctual attendance challenging. This pressure manifests as classroom behavioral issues such as distracted attention and diminished learning efficiency.

### 3.7 Analysis of the moderating effect of commuting method

This study examines the moderating effect of commuting methods on the relationship between commuting time and academic performance through stratified regression analysis, as presented in Table 8.

**Table 8:** Results of the moderation effect test for commuting methods.

Dependent variable	Chinese language score (M1)		Chinese language score (M2)		Mathematics score (M3)		Mathematics score (M4)		English language score (M5)		English language score (M6)	
	Beta	*P*	Beta	*P*	Beta	*P*	Beta	*P*	Beta	*P*	Beta	*P*
Commuting time	−0.046*** (0.007)	0.000	−0.063*** (0.007)	0.000	−0.038** (0.011)	0.001	−0.049*** (0.011)	0.000	−0.057*** (0.010)	0.000	−0.070*** (0.010)	0.000
Active Commuting (relative to “passive commuting”)	−0.087*** (0.455)	0.000	−0.089*** (0.454)	0.000	−0.096*** (0.714)	0.000	−0.097*** (0.714)	0.000	−0.116*** (0.659)	0.000	−0.118*** (0.658)	0.000
Commuting time × Active commuting			−0.066*** (0.014)	0.000			−0.040*** (0.021)	0.000			−0.049*** (0.020)	0.000
Control variable	Yes		Yes		Yes		Yes		Yes		Yes	
Time fixed effects	Yes		Yes		Yes		Yes		Yes		Yes	
*N*	8,496		8,496		8,498		8,498		8,489		8,489	
*R*-squared	0.021		0.025		0.027		0.029		0.062		0.064	
Adj *R*-squared	0.020		0.024		0.026		0.027		0.061		0.063	
*F*	14.273***		15.825***		18.394***		18.051***		43.107***		41.572***	

*P < 0.05; **P < 0.01; ***P < 0.001; values in parentheses indicate standard error (S.E.).

**Figure 2:**
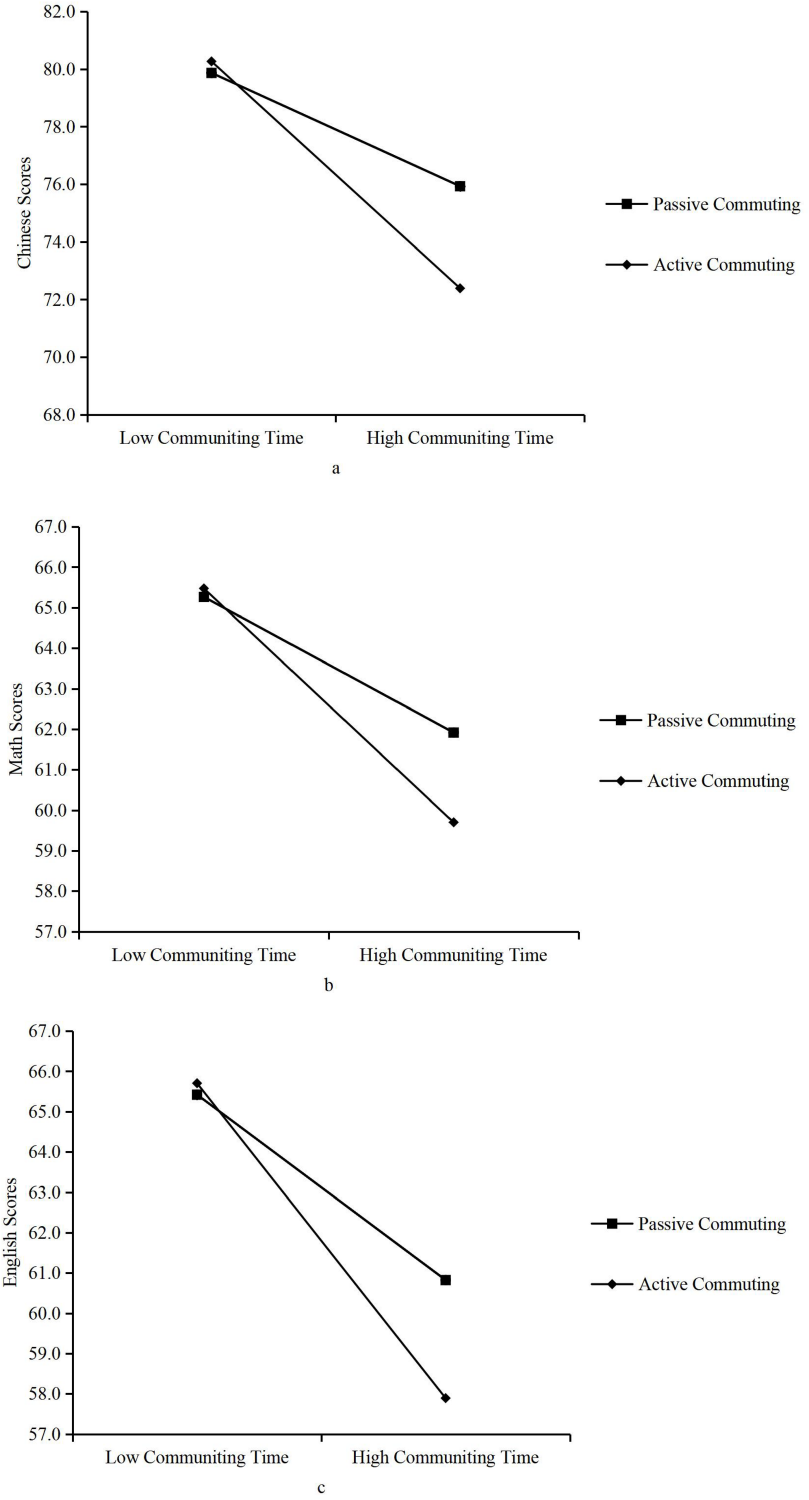
**(a)** Moderation effect slope of commuting mode on commuting time and Chinese language scores among students; **(b)** moderation effect slope of commuting mode on commuting time and mathematics performance among students; **(c)** moderation effect slope of commuting mode on commuting time and English grades among students.

Model 1 and Model 2 in Table 8 respectively demonstrate the moderating effects of commuting mode on the relationship between commuting time and students’ Chinese language scores. Model 1 shows that students who actively commute score 0.087 points lower than those who passively commute (*p* = 0.000 < 0.001). In Model 2, the interaction term between commuting time and active commuting significantly negatively predicted Chinese language scores (*α* = −0.066, *p* = 0.000 < 0.001). This indicates that, relative to passive commuting, active commuting exerts a significant positive moderating effect on the impact of commuting time on Chinese language scores. To further validate the moderating effect of commuting mode on the relationship between commuting time and Chinese language scores among students, this study employed a scatterplot with simple slope plots. As shown in Figure 2a, commuting time exhibits a significant negative predictive effect on Chinese language scores regardless of whether students choose active or passive commuting. However, compared to the passive commuting sample group, the negative correlation between commuting time and Chinese language scores is more pronounced in the active commuting sample group. This indicates that active commuting intensifies the negative impact of commuting time on student performance.

Model 3 and Model 4 in Table 8 examine the moderating effect of commuting mode on the relationship between commuting time and students’ math scores. Model 3 shows that longer commuting times correlate with lower math scores. Students who actively commute score 0.096 points lower in math than those who passively commute. Model 4 presents regression results for students’ math scores, accounting for commuting time, commuting mode, and their interaction. The regression coefficient for the interaction term between commuting time and active commuting on mathematics scores was −0.04, statistically significant at the 0.1% level. This indicates that commuting mode significantly moderates the relationship between commuting time and mathematics scores. As shown in Figure 2b, commuting time generally exerts a negative impact on mathematics scores. Compared to passive commuters, the negative correlation between commuting time and math scores is more pronounced among active commuters, suggesting that active commuting intensifies the detrimental impact of commuting time on math performance.

Model 5 and Model 6 in Table 8 examined the moderating effect of commuting mode on the relationship between commuting time and English grades among students. Model 5 revealed that commuting time significantly negatively predicted English grades (*β* = −0.059, *p* = 0.000 < 0.001); students who actively commuted scored 0.116 points lower than those who passively commuted (*p* = 0.000 < 0.001). In Model 6, the regression coefficient for the interaction term between commuting time and commuting mode was −0.049 (*p* = 0.000 < 0.001), statistically significant at the 0.1% level. This indicates that commuting mode significantly moderates the relationship between commuting time and English grades. As shown in Figure 2c, commuting time exhibits a significant negative predictive effect on English grades regardless of whether students choose active or passive commuting. However, the negative correlation between commuting time and English grades was more pronounced among active commuters than passive commuters, suggesting that active commuting intensifies the detrimental impact of commuting time on English performance. Through stratified regression and slope plots, research hypothesis H5 was not supported.

The adjustment effect of commuting methods indicates that active commuting does not mitigate the negative impact of commuting on students’ academic performance, which aligns with some findings by Jose Mora-Gonzalez and colleagues. Jose et al. studied 2,138 primary and secondary school students in Spain, including 489 primary school students and 1,649 middle school students, to analyze the relationship between active commuting and academic performance (Mora-Gonzalez et al., 2017). The study found that active commuting is significantly negatively correlated with the academic performance of primary school students but has no significant impact on middle school students. Active commuting exacerbates the negative impact of commuting time on students’ academic performance. This may stem from the lack of active commuting habits among local students, who primarily rely on parental transportation. Active commuting requires students to choose walking or cycling to school, demanding additional time and effort. Excessively long commutes inevitably increase students’ physical and psychological burdens. When academic pressure combines with commuting stress, exceeding students’ psychological and physiological limits, it inevitably intensifies psychological strain and anxiety, ultimately hindering academic progress. Although active commuting provides more exercise opportunities, the relationship between commuting activity levels and student academic performance follows an inverted U-shaped curve. Excessively long exercise durations during commutes may actually diminish their positive impact on academic performance (Xiang, 2021).

## 4 Discussion

This study aims to uncover the intrinsic relationship between commuting time and academic performance among students. Findings reveal that commuting time significantly impedes students’ performance in Chinese, mathematics, and English. Commuting time also indirectly impacts academic performance by reducing students’ learning engagement and increasing psychological fatigue. Furthermore, the study found that commuting mode significantly moderates the effect of commuting time on academic performance. Compared to passive commuting, active commuting intensifies the negative impact of commuting time on academic performance.

First, longer commutes are often associated with poorer academic performance among students, indicating that commuting consumes valuable energy and hinders their academic progress. This finding aligns with commuting stress theory. According to this theory, longer commutes increase students’ exposure to uncontrollable factors such as noise pollution, transportation breakdowns, and extreme weather conditions (Lutz et al., 2024). These uncontrollable factors subject students to multiple sensory stimuli during commutes, imposing significant mental and psychological stress. This stress often persists into classroom learning, ultimately hindering academic performance.

Second, learning engagement mediates the relationship between commuting time and academic performance among students. Research indicates that commuting time not only directly impedes academic performance but also indirectly affects academic performance by reducing learning commitment. Previous studies confirm that longer commutes correlate with lower learning commitment and delayed academic progress. This finding aligns with the Conservation of Resources Theory (COR) (Bon and Shire, 2022). COR posits that when resources are scarce or depleted, individuals proactively withdraw from activities that further consume cognitive and psychological resources to minimize resource loss. Excessive commuting forces students to sacrifice sleep to meet punctuality demands. Chronic sleep deprivation among students manifests as fatigue, poor mental states, and diminished sense of purpose and control over academic tasks. Ultimately, this weakens their engagement in high-challenge learning activities and teacher-student interactions. Research reveals that psychological exhaustion significantly mediates the relationship between commuting duration and academic performance. Prolonged commutes demand greater behavioral, cognitive, and emotional effort from students. When these efforts are expended without adequate physiological and psychological recovery, fatigue ensues. Fatigue is a hallmark of psychological exhaustion, manifesting through a range of stress responses including depression and anxiety. Students experiencing these stressors find it increasingly difficult to maintain satisfactory learning behaviors in the classroom (De Reuver and Biron, 2023).

## 5 Implications

This study reveals the interrelationship between commuting time and students’ academic performance, learning engagement, and psychological fatigue, providing theoretical support for improving real-world educational environments. Commuting time acts as an obstacle to academic performance development among students, mediating the relationship between learning engagement and psychological fatigue, while also exhibiting a moderating effect based on commuting mode. This indicates that school administrators and policymakers need to allocate more resources to school district planning, student boarding, school bus services, and psychological counseling.

First, China must maintain a dense school network. While school consolidation continues, the number of institutions should be strategically allocated: avoiding the merger of all small-enrollment primary and secondary schools. Particularly in remote and sparsely populated areas, educational facilities or teaching points should be preserved. This ensures students from economically disadvantaged and rural families can attend nearby schools, minimizing unnecessary commuting time.

Second, schools should provide ample bus transportation and boarding services. In China, voluntary commuting by students significantly increases the negative impact of commuting time on academic performance. Therefore, schools can lease or purchase dedicated vehicles to transport students, minimizing the adverse effects of voluntary commuting. Schools can also expand dormitory facilities to offer boarding services for students with longer commutes.

Finally, schools should provide psychological counseling and academic support to alleviate mental fatigue caused by excessive commuting, thereby enhancing students’ engagement and initiative in learning. Teachers can offer after-school tutoring and guide students to form peer study groups to assist those whose academic performance has declined due to commuting challenges. Schools may also consider delaying morning class start times to provide a buffer period for students with longer commutes.

## 6 Conclusion

This study provides valuable insights into how commuting time, learning engagement, and psychological fatigue predict academic performance among students. Commuting time emerges as a key factor in the decline of academic performance. Psychological fatigue and learning engagement exert a significant mediating effect between commuting time and academic performance. Commuting mode significantly moderates the impact of commuting time on academic performance—active commuting intensifies the negative effect of commuting time on academic performance compared to passive commuting.

Beyond its theoretical value, this study holds substantial practical significance. Effective interventions within educational settings and activities can mitigate the negative impact of commuting time as a stressor on students. Such measures contribute to creating more equitable and supportive learning environments while empowering students’ psychological resilience, enhancing learning engagement, and improving academic performance.

## 7 Limitations

This study examines the impact of commuting time on academic performance among students, but certain limitations exist regarding data, research subjects, and analytical methods. First, the data used in this study are from the 2014–2015 longitudinal cohort. This dataset did not collect information on the distance students commute from home to school, making it impossible to include commuting distance as a control variable. This omission may introduce bias into the estimated effect of commuting time on academic performance. Second, the study population consisted solely of eighth-grade students. This precludes testing the effects of commuting time on academic performance across different grade levels, potentially limiting the generalizability of the findings. Finally, due to data confidentiality, the 2014–2015 longitudinal data obscured the geographic location of schools, preventing the inclusion of region as a fixed effect in the regression analysis.

## Data Availability

The original contributions presented in the study are included in the article/supplementary material, further inquiries can be directed to the corresponding author/s.
